# Supplementary material for: Quantum Transport in Nitrogen‐Doped Nanoporous Graphenes
Source: Small. 2025 Nov 10;21(51):e08850. doi: 10.1002/smll.202508850 (PMC12723339; doi:10.1002/smll.202508850)
Supplement: Supplementary file 1 — Supporting Information [file SMLL-21-e08850-s001.pdf]

NANO · MICRO
small

Supporting Information

for *Small*, DOI 10.1002/smll.202508850

Quantum Transport in Nitrogen-Doped Nanoporous Graphenes

Gaetano Calogero, Isaac Alcón, Alan E. Anaya Morales, Nick Papior, Pol Febrer, Aron W. Cummings, Miguel Pruneda, Stephan Roche and Mads Brandbyge*

Supporting Information

Quantum transport in nitrogen-doped nanoporous graphenes

Gaetano Calogero, Isaac Alcón, Alan Morales, Nick Papior, Pol Febrer, Aron W. Cummings, Miguel Pruneda, Stephan Roche and Mads Brandbyge*

Supporting Information

Quantum transport in nitrogen-doped nanoporous graphenes

Gaetano Calogero[1†], Isaac Alcón[2,3†*], Alan Morales[4], Nick Papior[5], Pol Febrer[2], Aron W. Cummings[2], Miguel Pruneda[2,6], Stephan Roche[2,7] and Mads Brandbyge[4]

[1]*National Research Council, Institute for Microelectronics and Microsystems (CNR-IMM), Zona Industriale, Strada VIII, 5, 95121 Catania, Italy*

[2]*Catalan Institute of Nanoscience and Nanotechnology (ICN2), CSIC and BIST, Campus UAB, Bellaterra, 08193 Barcelona, Spain*

[3]*Institute of Theoretical and Computational Chemistry, Department of Materials Science and Physical Chemistry, Universitat de Barcelona, C/ de Martí i Franquès, 1-11, Les Corts, 08028, Barcelona, Spain*

[4]*Department of Physics, Technical University of Denmark, DK-2800 Kongens Lyngby, Denmark*

[5]*Computing Center, Technical University of Denmark, DK-2800 Kongens Lyngby, Denmark*

[6]*Nanomaterials and Nanotechnology Research Centre (CINN), CSIC, Avenida de la Vega 4-6. 33940, El Entrego, Spain*

[7]*ICREA, Institució Catalana de Recerca i Estudis Avançats, 08070 Barcelona, Spain*

[†]equally contributed

*Corresponding author: ialcon@ub.edu

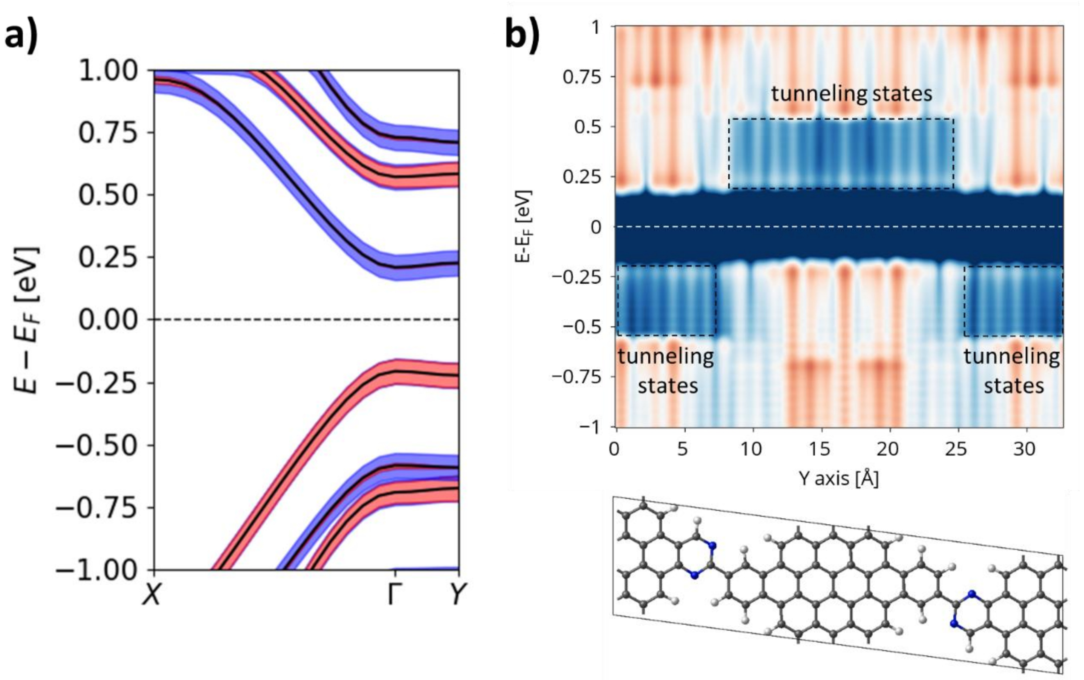

Figure S1. a) GNR-resolved fat band structure (cGNR: red; nGNR: blue) of hNPG in the neutral state. b) Local density of states (LDOS) along the y-axis, showing that valence (conduction) states are mainly located in the cGNR (nGNR), and that tunnelling states exist at all energies beyond the band edges. The colors are in a log scale, with dark red (blue) indicating a high (low) LDOS. The Fermi level (E_F) is indicated with a dashed white line. The hNPG atomic structure is provided in the bottom panel for a one-to-one comparison with the LDOS plot.

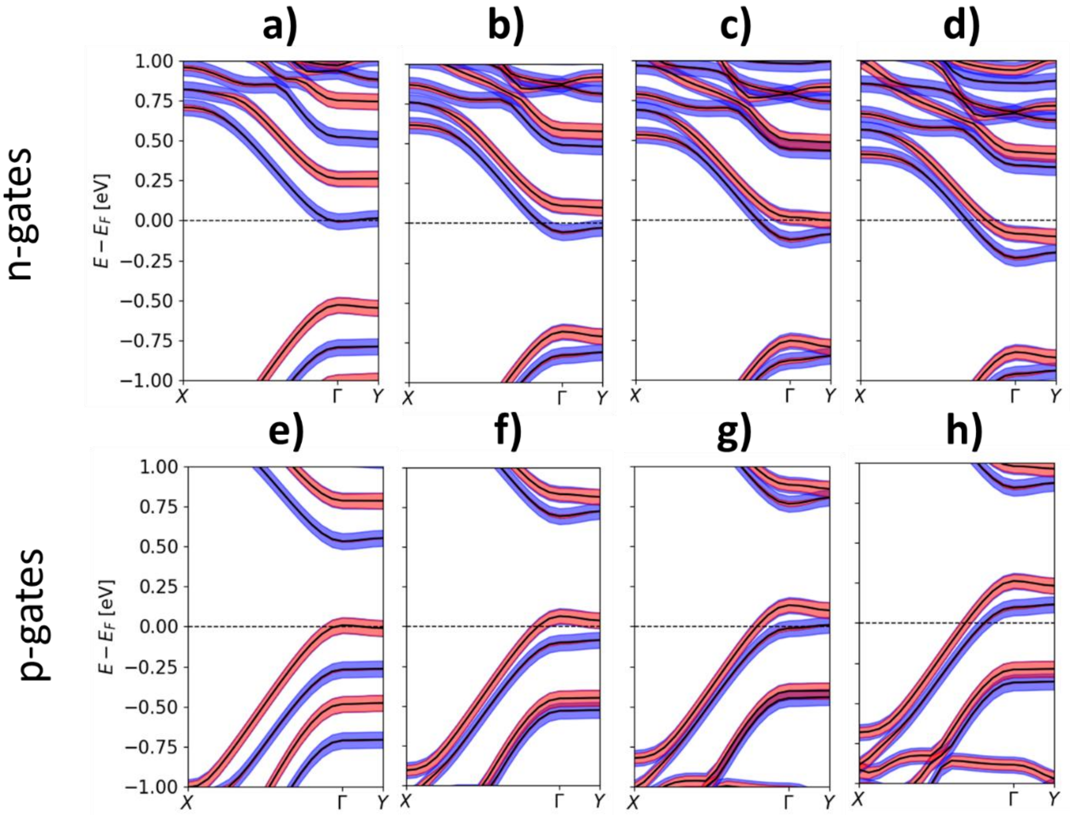

Figure S2 GNR-resolved fat band structures (cGNR: red; nGNR: blue) of hNPG for n-gates a) +0.1 e/cell (+0.36 · 10^{13} e/cm^2), b) +0.25 e/cell (+0.89 · 10^{13} e/cm^2), c) +0.5 e/cell (+1.79 · 10^{13} e/cm^2), d) +1.0 e/cell (+3.58 · 10^{13} e/cm^2), and p-gates e) -0.1 e/cell (-0.36 · 10^{13} e/cm^2), f) -0.25 e/cell (-0.89 · 10^{13} e/cm^2), g) -0.5 e/cell (-1.79 · 10^{13} e/cm^2), h) -1.0 e/cell (-3.58 · 10^{13} e/cm^2).

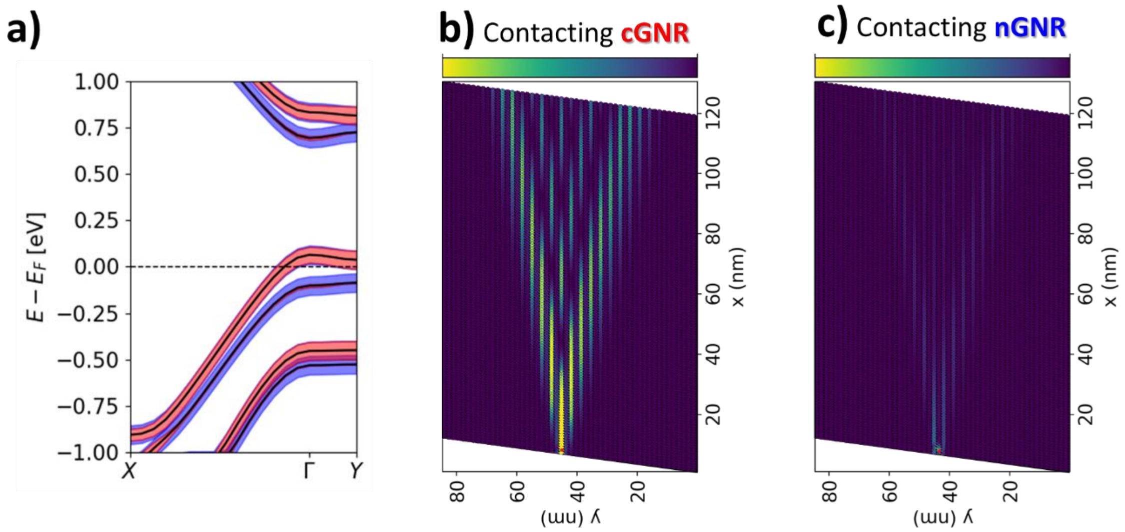

Figure S3. a) GNR-resolved fat band structure (cGNR: red; nGNR: blue) of hNPG for -0.25 e/cell gating (-0.89 · 10^{13} e/cm^2) and corresponding bond transmission maps of a ~85 x 122 nm^2 sample upon locally contacting b) a cGNR or c) a nGNR at the bottom part of the sample (see small red dot). The color bars in the bond transmission maps range from 0 to 0.045. The Hamiltonian of these large-scale samples has been obtained by taking p_z orbitals from the DFT Hamiltonian (see Methods for details).

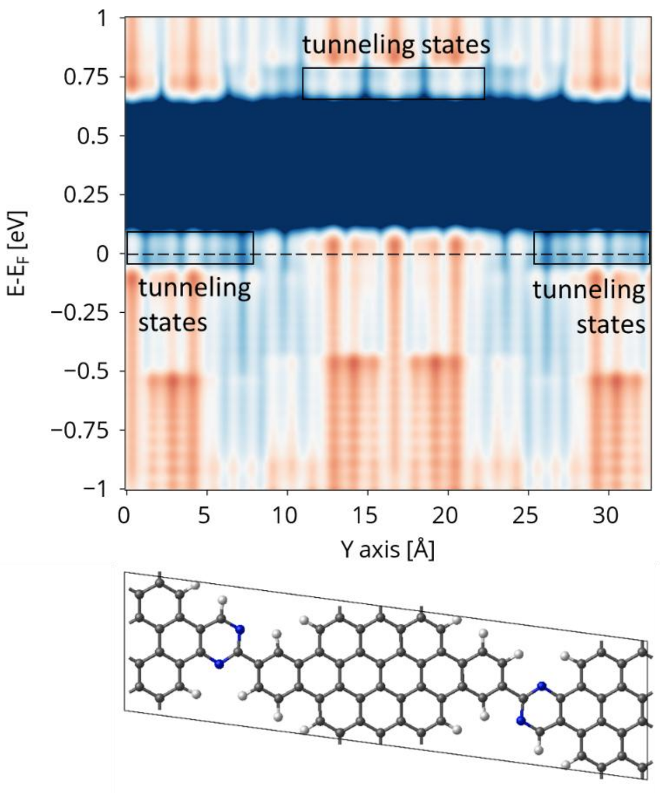

Figure S4. LDOS along the y-axis for the -0.25 e/cell gated hNPG ($-0.89 \cdot 10^{13}$ e/cm^2). The colors are in a log scale, with dark red (blue) indicating a high (low) LDOS. E_F is indicated with a dashed black line. The corresponding band structure is shown in Figure S3a. The hNPG atomic structure is provided in the bottom panel for a one-to-one comparison with the LDOS plot.

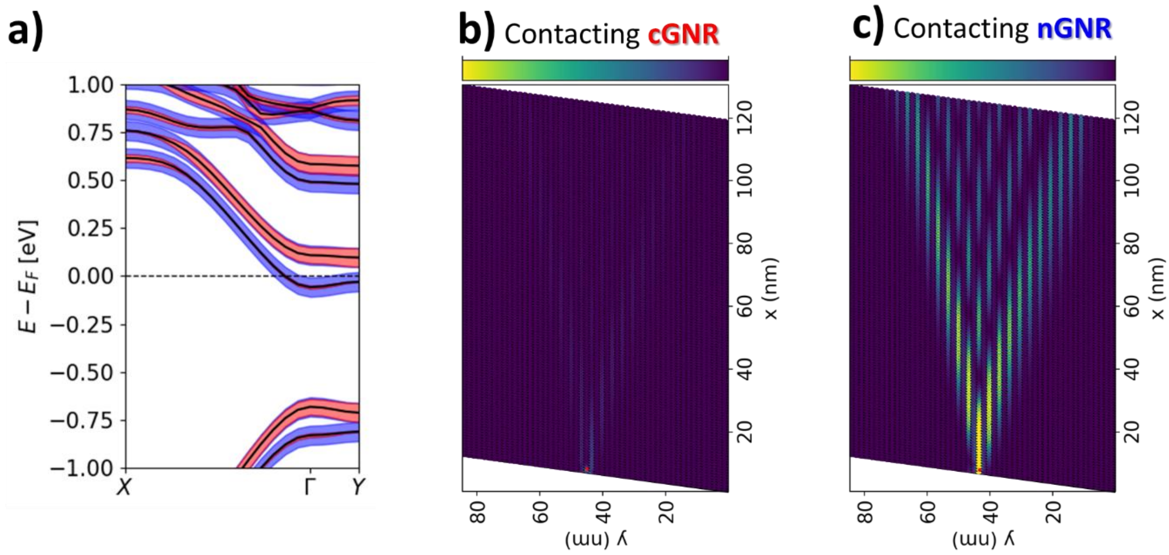

Figure S5. a) GNR-resolved fat band structure (cGNR: red; nGNR: blue) of hNPG for +0.25 e/cell gating ($+0.89 \cdot 10^{13}$ e/cm^2) and corresponding bond transmission maps of a ~85 x 122 nm^2 sample upon locally contacting b) a cGNR or c) a nGNR at the bottom part of the sample (see small red dot). All color bars in the bond transmission maps range from 0 to 0.045. The Hamiltonian of these large-scale samples has been obtained by taking p_z orbitals from the DFT Hamiltonian (see Methods for details).

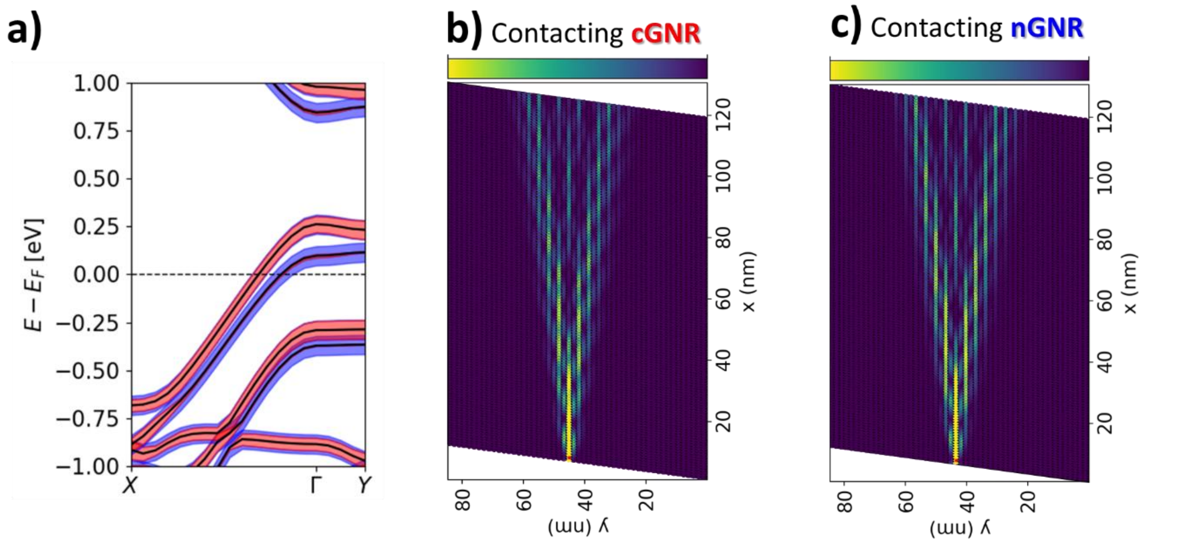

Figure S6. a) GNR-resolved fat band structure (cGNR: red; nGNR: blue) of hNPG for -1.0 e/cell gating (-3.58 · 10^{13} e/cm^2) and corresponding bond transmission maps of a ~85 x 122 nm^2 sample upon locally contacting b) a cGNR or c) a nGNR at the bottom part of the sample (see small red dot). All color bars in the bond transmission maps range from 0 to 0.045. The Hamiltonian of these large-scale samples has been obtained by taking p_z orbitals from the DFT Hamiltonian (see Methods for details).

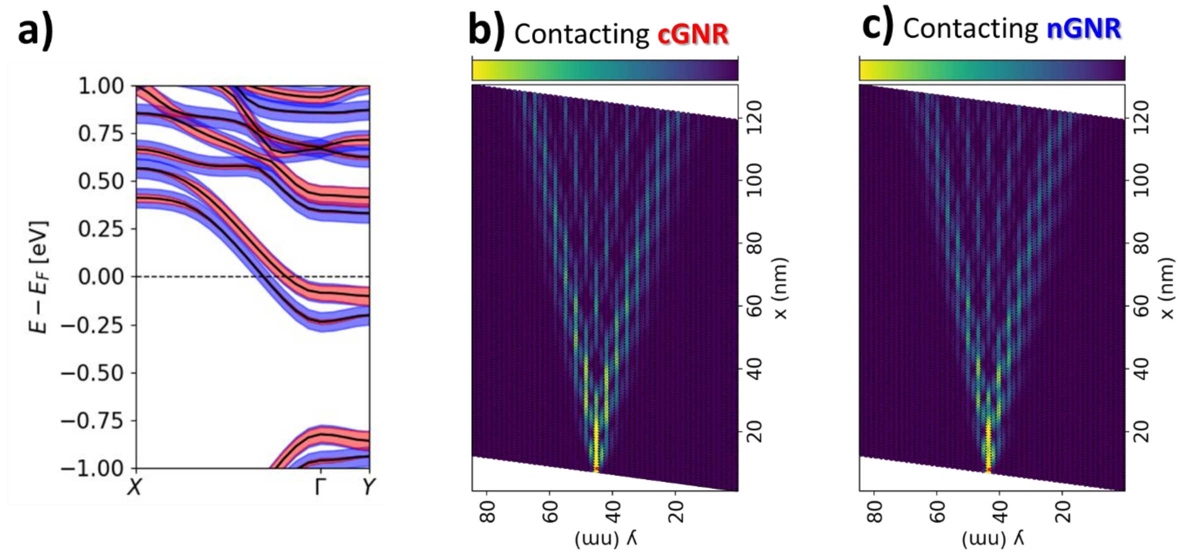

Figure S7. a) GNR-resolved fat band structure (cGNR: red; nGNR: blue) of hNPG for +1.0 e/cell gating (+3.58 · 10^{13} e/cm^2) and corresponding bond transmission maps of a ~85 x 122 nm^2 sample upon locally contacting b) a cGNR or c) a nGNR at the bottom part of the sample (see small red dot). All color bars in the bond transmission maps range from 0 to 0.045. The Hamiltonian of these large-scale samples has been obtained by taking p_z orbitals from the DFT Hamiltonian (see Methods for details).

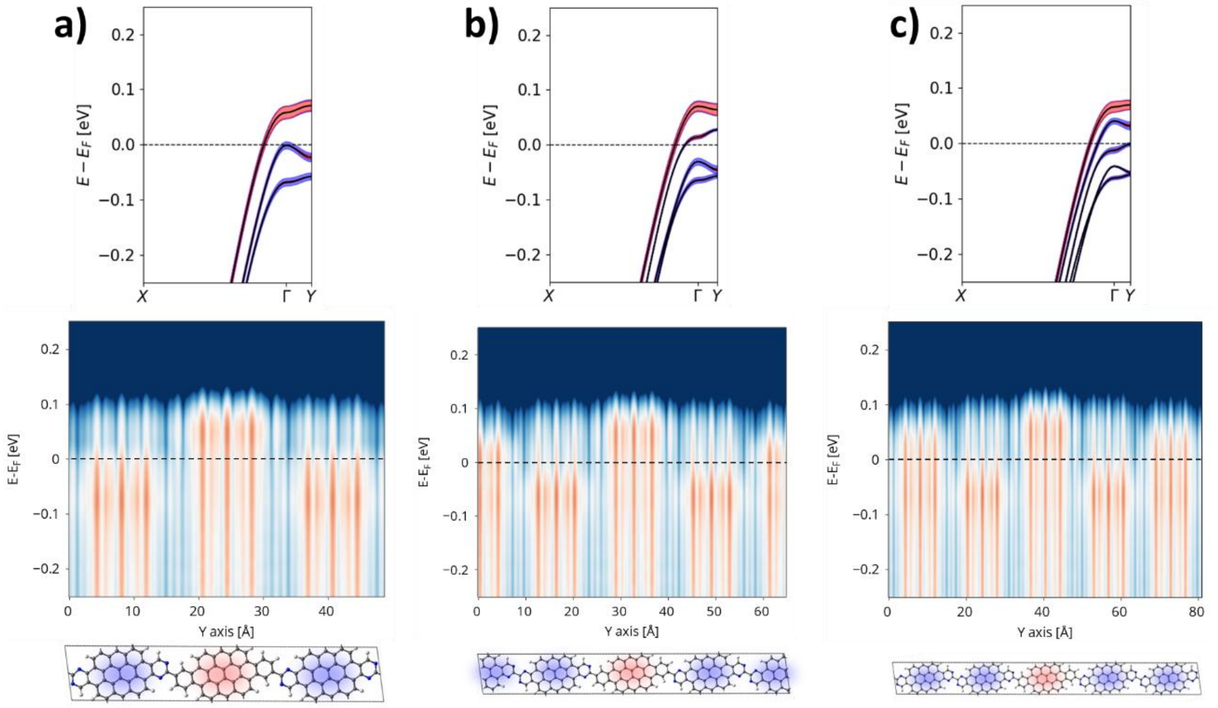

Figure S8. Electronic analysis of the -0.90 · 10^{13} e/cm^2 p-gated a) hNPG-1,2, b) hNPG-1,3 and c) hNPG-1,4 heterostructures. Top panels display the GNR-resolved fat band structure (cGNR: red; nGNR: blue) for each case. Middle panels display the y-resolved LDOS, including each corresponding heterostructure geometry at the bottom. The colors are in a log scale, with dark red (blue) indicating a high (low) LDOS. E_F is indicated with a dashed black line.

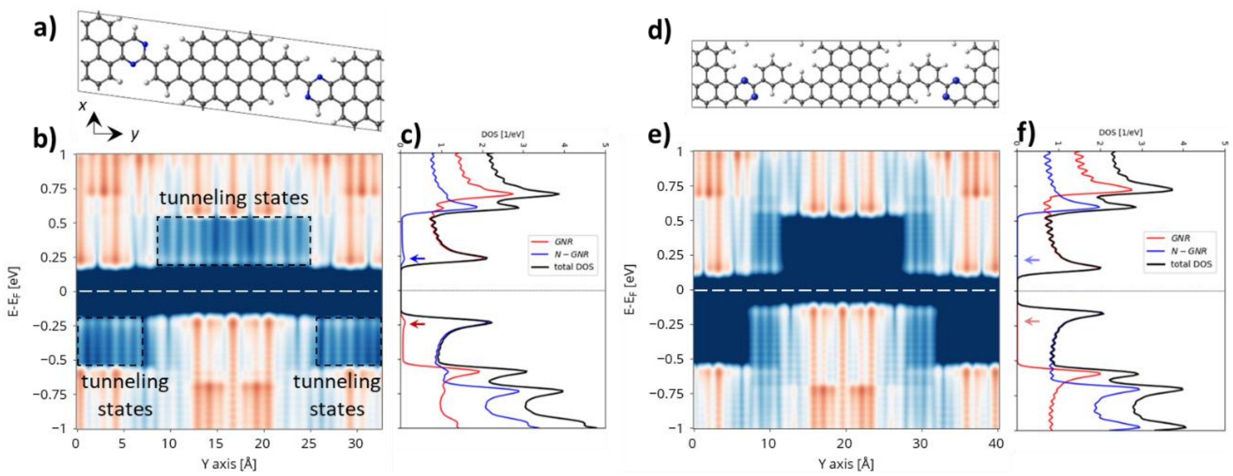

Figure S9. Comparison between the atomic structures, y-resolved LDOS and partial density of states (PDOS) for hNPG (a-c) and meta-hNPG (d-f). In panels c) and f) the cGNR and nGNR contributions are indicated with red and blue curves, respectively, with the total DOS shown in black. In the LDOS plots (b and e) the colors are in a log scale, with dark red (blue) indicating a high (low) LDOS. E_F is indicated with a dashed white line.

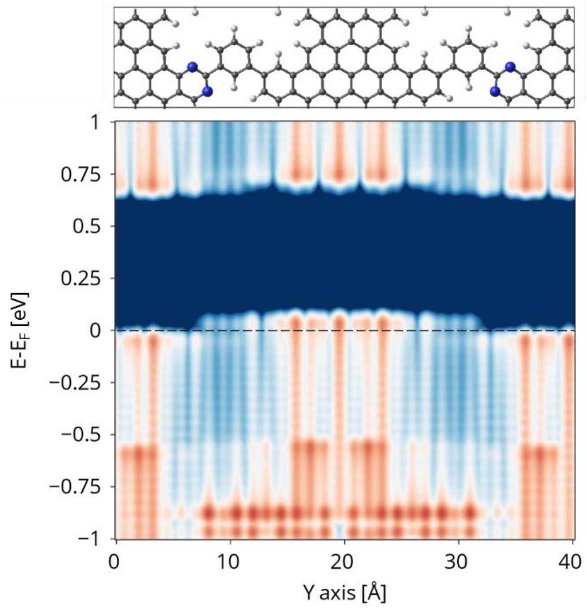

Figure S10. y-resolved LDOS for the -0.72 · 10^{13} e/cm^2 p-gated meta-hNPG (-0.25 e/cell). The colors are in log scale, with dark red (blue) indicating a high (low) LDOS. E_F is indicated with a dashed black line. The meta-hNPG atomic structure is provided in the top panel for a one-to-one comparison with the LDOS plot.

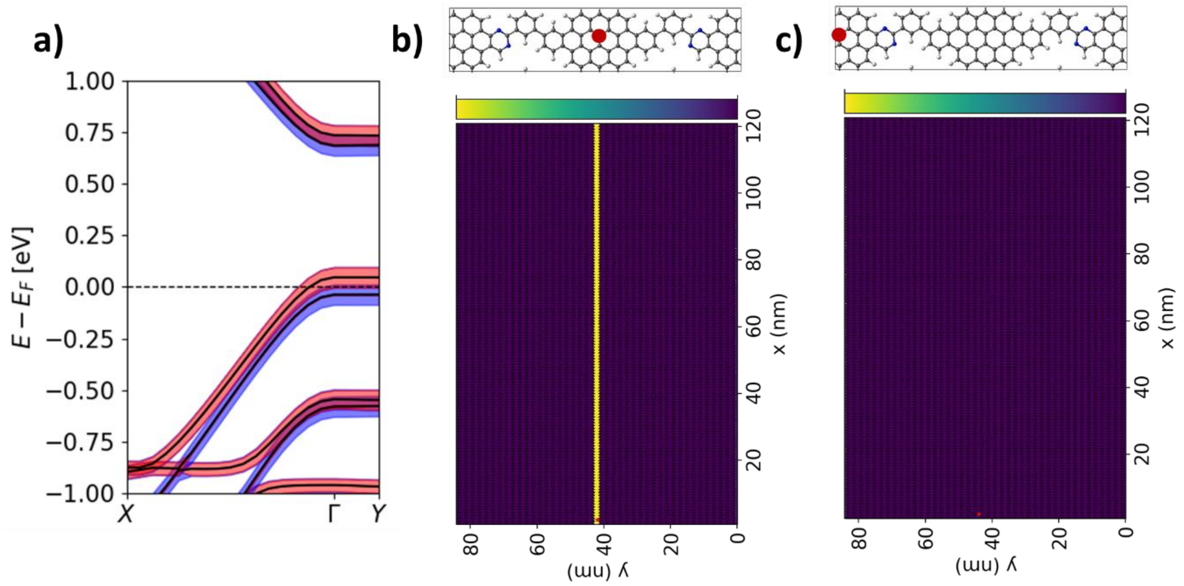

Figure S11. a) GNR-resolved fat band structure (cGNR: red; nGNR: blue) of meta-hNPG for -0.25 e/cell gating (-0.72 · 10^{13} e/cm^2) and corresponding bond transmission maps of a ~85 x 122 nm^2 sample upon locally contacting b) a cGNR or c) a nGNR at the bottom part of the sample (see small red dot. All color bars in the bond transmission maps range from 0 to 0.045. The Hamiltonian of these large-scale samples has been obtained by taking p_z orbitals from the DFT Hamiltonian (see Methods for details). Top panels show the atomic structure of meta-hNPG, indicating with a red sphere the injection point in each case.

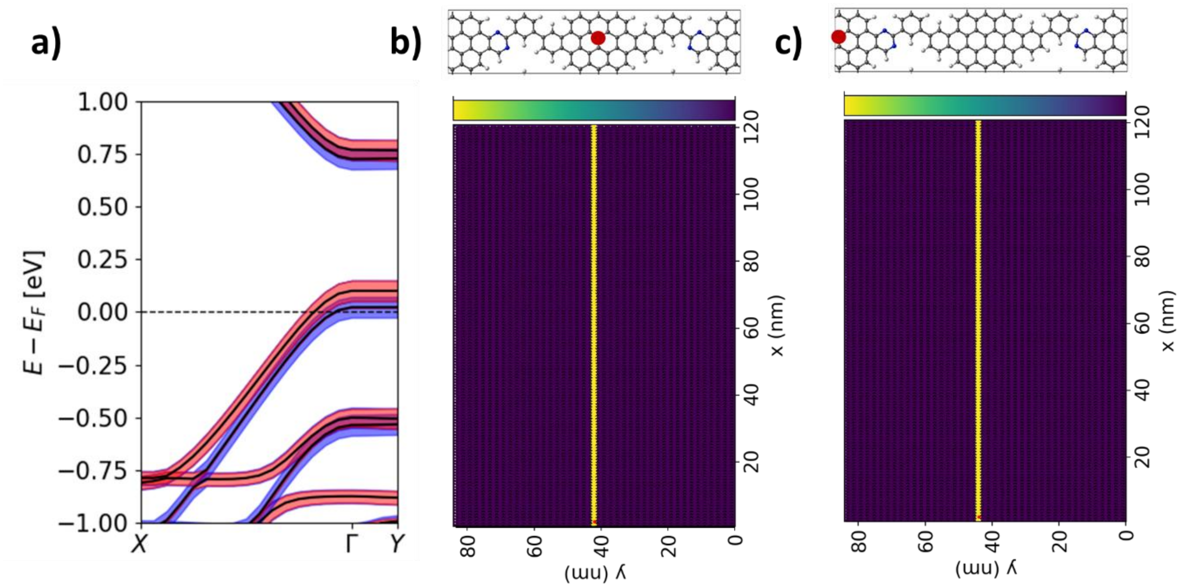

Figure S12. a) GNR-resolved fat band structure (cGNR: red; nGNR: blue) of meta-hNPG for -0.5 e/cell gating (-1.44 · 10^{13} e/cm^2) and corresponding bond transmission maps of a ~85 x 122 nm^2 sample upon locally contacting b) a cGNR or c) a nGNR at the bottom part of the sample (see small red dot). All color bars in the bond transmission maps range from 0 to 0.045. The Hamiltonian of these large-scale samples has been obtained by taking p_z orbitals from the DFT Hamiltonian (see Methods for details). Top panels show the atomic structure of meta-hNPG, indicating with a red sphere the injection point in each case.

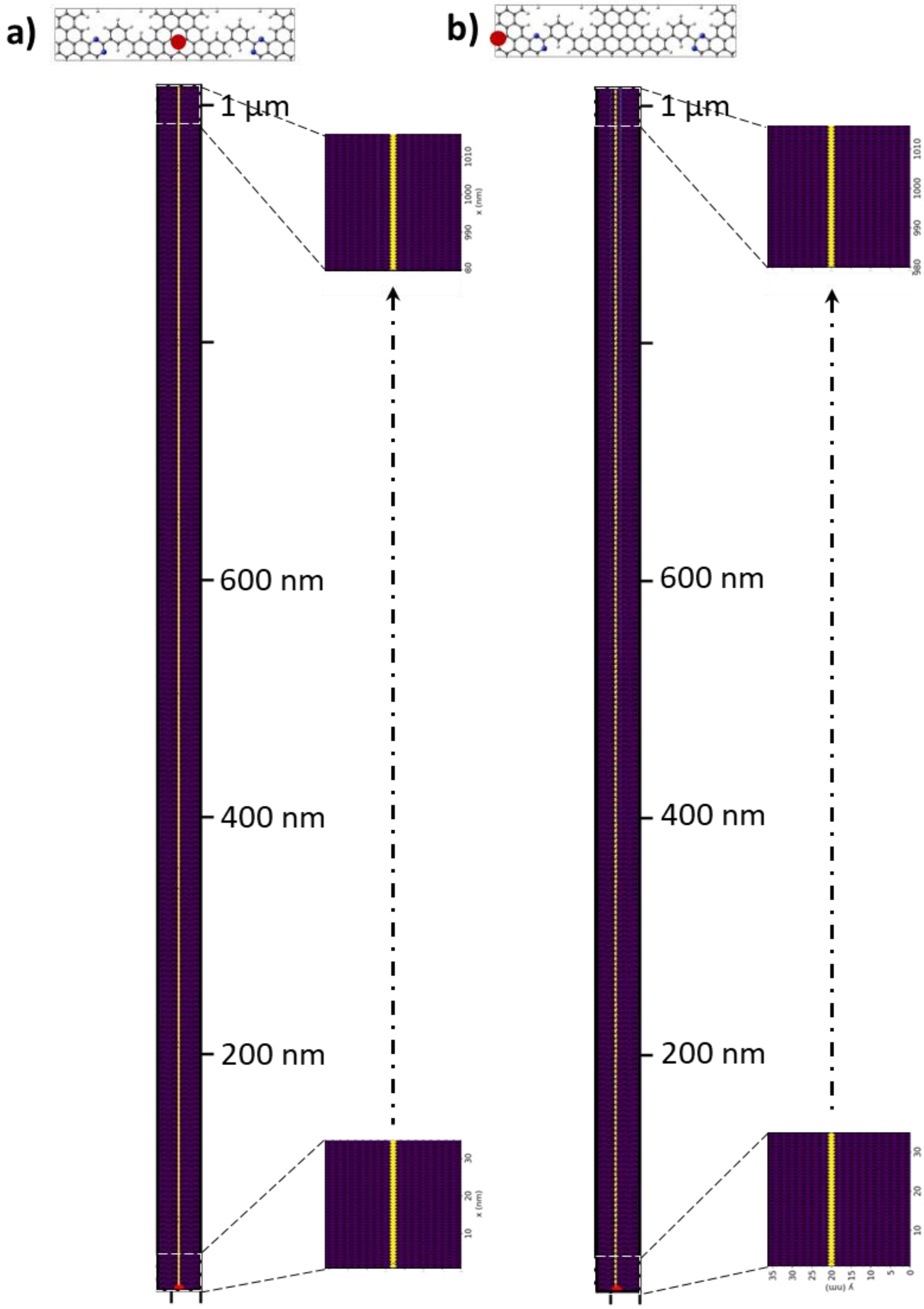

Figure S13. Bond transmission maps of a -0.5 e/cell gated ($-1.44 \cdot 10^{13}$ e/cm^2) ~36 x 1017 nm^2 meta-hNPG sample (968,760 atoms) upon locally contacting the bottom region of a) the central cGNR or b) the central nGNR (see red dots in the atomic structure in the top panel and in the device bottom region). A zoomed visual of the bond transmission maps is provided for the bottom and top regions, to highlight the level of collimation 1 µm away from the injection point. All color bars range from 0 to 0.045.

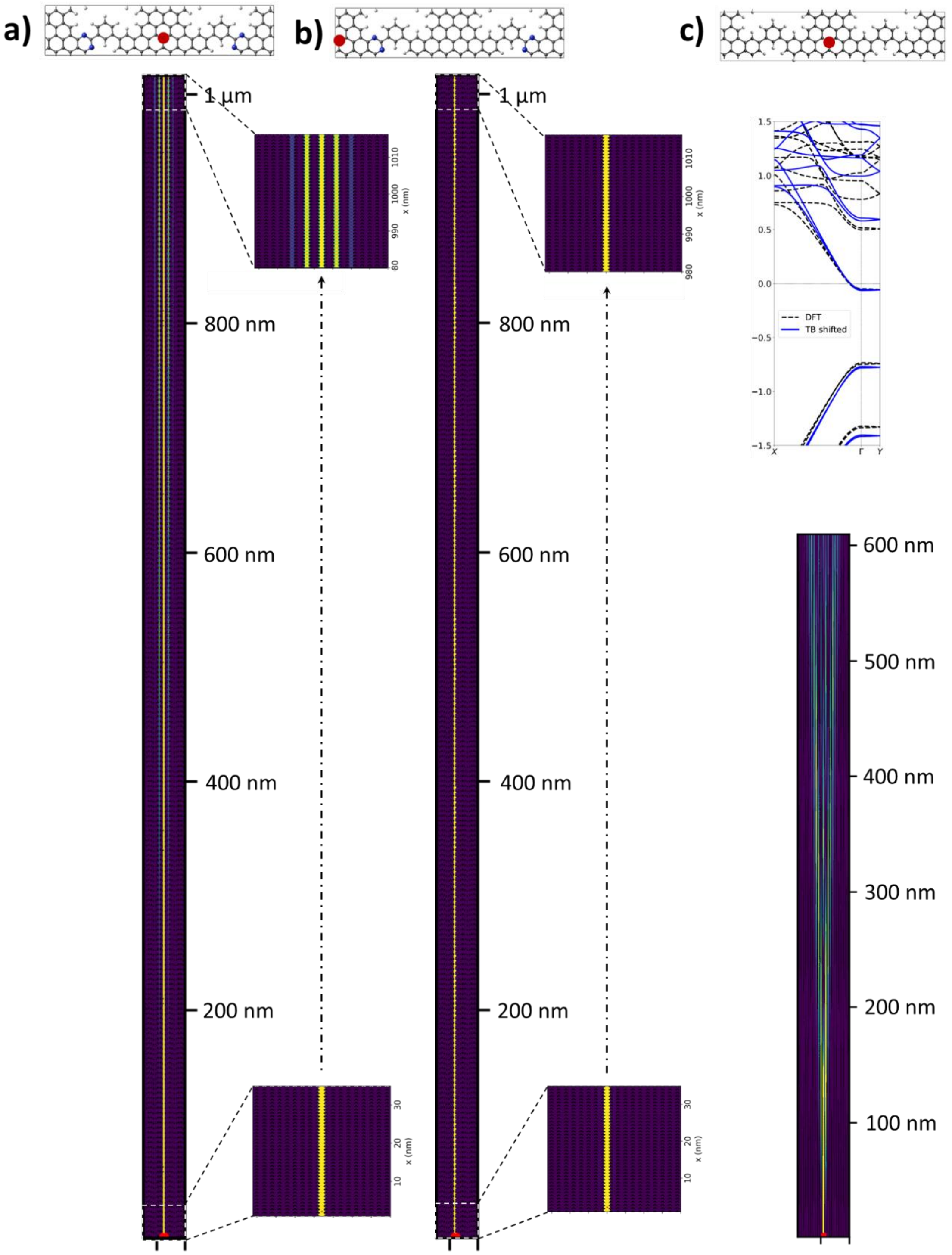

Figure S14 Bond transmission maps of a +0.5 e/cell gated (+1.44 · 10^{13} e/cm^2) ~36 x 1017 nm^2 meta-hNPG sample (968,760 atoms) upon locally contacting a) a cGNR or b) a nGNR at the bottom of the device (see small red dot). All color bars in the bond transmission maps range from 0 to 0.045. The injection point is shown as a red sphere in the atomic structures provided at the top. For both cases, a zoomed visual of the bond transmission map is provided for the bottom and top regions of the device, to highlight their different behavior far away from the source. c) Bond transmission maps of a +0.5 e/cell gated (-1.42 · 10^{13} e/cm^2) ~45 x 610 nm^2 meta-NPG sample (710,424 atoms) upon locally injecting at the bottom of the device (see small red dot). All color bars range from 0 to 0.045. The corresponding optimized atomic structure and band structure are provided in the top panels. Both DFT and DFT-parameterized TB bands are shown, with aligned conduction band edges (see Methods).

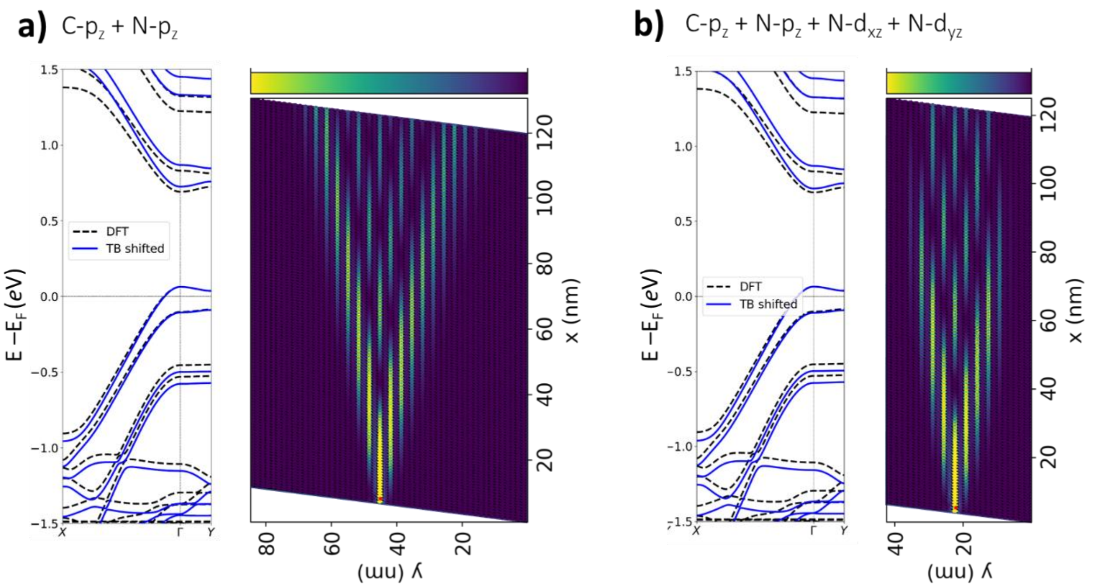

Figure S15 Comparison between band structures and bond transmission maps of a -0.25 e/cell p-gated hNPG system (-0.89 x 10^{13} e/cm^3) obtained by parameterizing the tight-binding Hamiltonian using DFT-converged Hamiltonian elements associated with (a) C-p_z and N-p_z or (b) C-p_z, N-p_z, N-d_{xz} and Nd_{yz} orbitals. All color bars in the bond transmission maps range from 0 to 0.045.

Supplementary Note S1

To validate the model based on coupled discrete differential equations (DDE), we compare the field intensity obtained from a parameterized model with bond-resolved transmissions obtained with the TB-GF methodology. The parametrization for the propagation constant (α) was set to:

$$\alpha = \frac{U}{\overline{dE/dk}} = \frac{|\overline{\epsilon_C} - \overline{\epsilon_N}|}{\overline{dE/dk}}.$$

This provides a good estimate of the model parameters of the model and establishes a connection between the propagation along the waveguides in the DDE model with the difference in the average p_z on-site potentials of cGNRs/nGNRs ($|\overline{\epsilon_C} - \overline{\epsilon_N}|$), normalized by the average band slope of the conduction band (group velocity of the conducting electrons, $\overline{dE/dk}$). For an n-doped hNPG device (0.36 · 10^{13} e/cm^2), we get $U = 0.248\ eV$ and an average band slope of 3.81 $eV/$Å. With this, $\alpha = 0.065$ Å^{-1}was obtained. We set $\kappa = 0.0137$ Å^{-1} from[1]

$$\kappa = \frac{|k_1 - k_0|}{4},$$

with k_0 and k_1 being the momenta associated with the first two conduction (or valence) linear bands dispersing along the Γ-X high-symmetry path of the Brillouin zone, evaluated at the injection energy E of $E - E_F = 0.1\ eV$. The TB-GF device used for comparison consists of an n-gated hNPG device (0.36 · 10^{13} e/cm^2) of 68.9 nm × 103 nm with 252,000 atoms, with the injection site located on a nGNR. As shown in Figure S16, we find good qualitative agreement between the field intensity propagation of the DDE model and the large-scale TB-GF calculations. The latter exhibits faster oscillations, attributed to intra-unit-cell hopping processes, which are not captured by the coarse-grained nature of our effective model (Figure S17b). Additionally, the amplitude of the oscillations is larger in the TB-GF results (Figure S17b), likely due to local variations in the on-site p_z orbital energies along the GNRs, as well as different atomic sublattices in the hNPG unit-cell (Figure S17a).

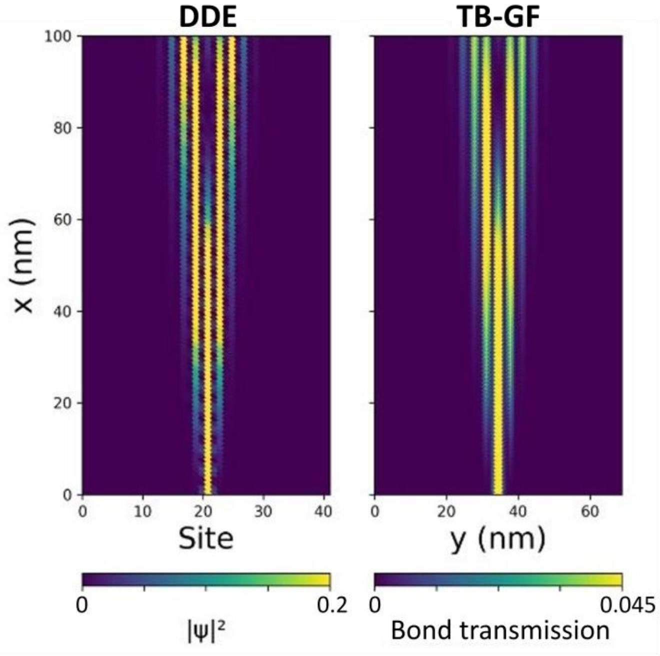

Figure S16. Comparison between the spatial intensity of waveguide modes from the DDE model and the bond transmission of large-scale TB-GF transport calculations for an n-gated hNPG device (0.36 · 10^{13} e/cm^2) contacted on an nGNR. Here α=0.065 Å^{-1} and κ=0.014 Å^{-1} (see Methods).

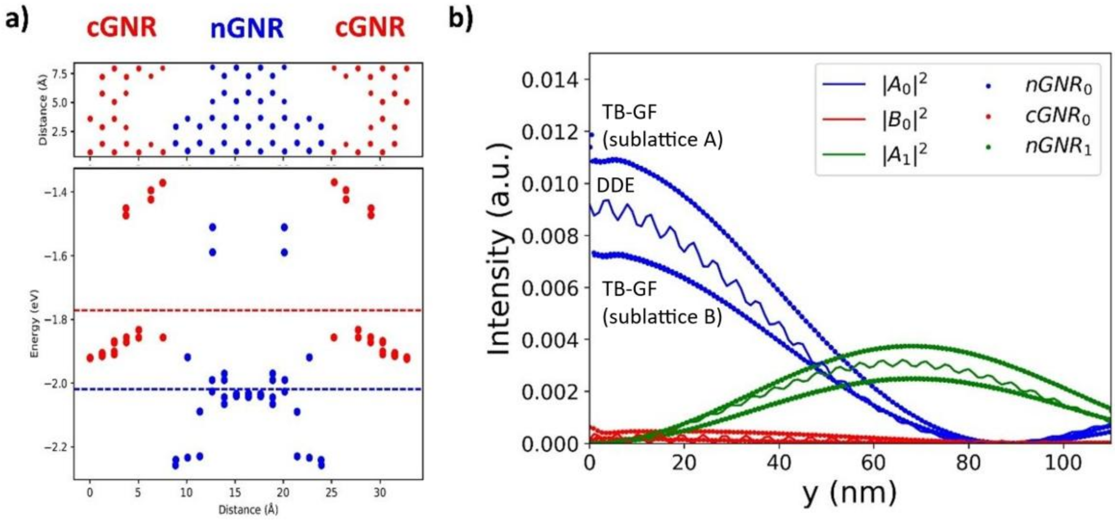

Figure S17. Parametrization of the DDE model for an n doped hNPG (0.36 · 10^{13} e/cm^2). (a) The top panel shows the hNPG structure from the parameter-free TB. The coloring of the atoms corresponds to the type of subsystem they belong to, with cGNR/nGNR being red/blue. The carbon p_z onsite energies are shown in the bottom panel. The dashed lines represent the average for each GNR used to determine α. (b) Comparison between the waveguide mode intensity from the DDE model (line) and the atom transmission from TB-GF calculations at the center of the GNRs (dotted) for the first three GNRs. In the TB-GF case, two dotted lines for each GNR illustrate the contributions from the two graphene sublattices. The waveguide mode intensity from the DDE model was scaled for comparison. The injection point in the TB-GF calculations was on an nGNR, which is conducting for the gating considered here.

Finally, we would like to note that the DFT calculations, by which the DDE model has been based upon, do not include electron-electron correlation effects, which have been shown to potentially reduce the band gap of carbon nanomaterials.[2] However, as demonstrated in that theoretical study, as long as the materials band gap is much larger than kBT (as in our case), electron-electron scattering should remain low, and so our parametrized α and κ values should not qualitatively change.

Robustness against random variations in the effective parameter α, up to 0.05 α, at individual ribbons shows that the interference pattern persists with statistic disorder for instance from thermal fluctuations.

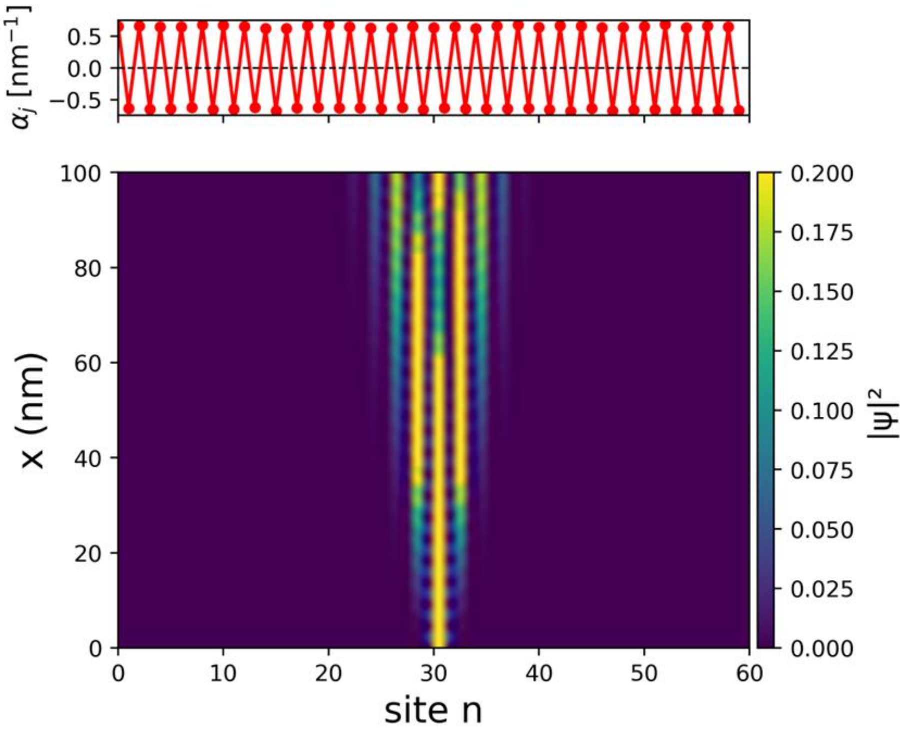

Figure S18. Spatial intensity of waveguide modes from the DDE model under random variations. Here $\alpha_j=(-1)^j\,0.065+\delta_j$ Å^{-1} and $\kappa=0.014$ Å^{-1} (see Methods).

Bibliography

(1) Calogero, G.; Papior, N. R.; Kretz, B.; Garcia-Lekue, A.; Frederiksen, T.; Brandbyge, M. Electron Transport in Nanoporous Graphene: Probing the Talbot Effect. *Nano Letters* **2018**, *19* (1), 576–581. https://doi.org/10.1021/acs.nanolett.8b04616.

(2) Valli, A.; Fabian, T.; Libisch, F.; Stadler, R. Stability of Destructive Quantum Interference Antiresonances in Electron Transport through Graphene Nanostructures. *Carbon* **2023**, *214*, 118358. https://doi.org/10.1016/j.carbon.2023.118358.
